# Extract from an Article on the Comparative Pathology of Post-partum Hemorrhage

**Published:** 1854-10

**Authors:** John Bremner

**Affiliations:** Surgeon


*Extract from an Article on the Comparative Pathology of Post-partum Hemorrhage.* By John Bremner, Surgeon.

Previous to having obtained a more intimate acquaintance with the anatomy of the gravid uterus in the inferior creation, and the experience of Mr. B. (Barlow, assistant Professor of the Edinburg Veterinary College) to the contrary, I had been inclined to imagine that the absence of post-partum discharges of blood depended in a great measure on a more effective state of uterine contraction than was generally met with in the case of woman; and would consequently be found of more frequent occurrence amongst domesticated animals whose muscular system had become relaxed from confinement to the stall before the termination of gestation, than others roaming at greater freedom, or altogether in the wild and migratory state. Laying aside the fact, however, that in the cow, at least, a period varying from eight or nine hours to several days is sometimes required for the discharge of the secundines, an inspection of the structure of each of these apart serves fully to dispel all doubt on the subject, and to assnre us that no such casuality can take place in the healthy and complete state of the organs and their appendages. A question, however, of no trifling importance here presents itself for our consideration.

As post-partum hemorrhage is known from time to time to occur amongst quadruped animals, occasionally in such profusion as to prove destructive to life—What are the circumstances under which it has been found to have taken place? The experience and testimony of Mr. B. on this department of the inquiry seem most decisive; he commences with stating, "I have not any recollection of a case of hemorrhage after delivery, wherein this (the delivery) was effected by the animal herself."

After making allusion to the difference of opinion entertained by different veterinary surgeons on this subject—which he has no hesitation whatever in ascribing to the imperfect manner in which post-mortem examinations are conducted, frequently to their entire neglect—he writes as follows:—"My own practice, which has been very extensive, goes to show that in *every case* where post-partum hemorrhage in domestic animals has terminated fatally, and wherein careful post-mortem dissections have been made, a visible lesion, rent, or wound, has been found in the uterine surface. In no case do I remember fatal hemorrhage to have occurred without injury of the placenta, notwithstanding that almost every fatal case has been minutely examined into for many years past." In a succeeding part of his communication he again states:—"I have often known death follow, apparently as the result of this cause (placental hemorrhage), but on dissection have always found some rent, or lesion of surface, in which vascular trunks were involved;" and immediately adds, "I have seen cases, but not very numerous, in which hemorrhage took place after delivery, and was overcome by treatment. In these, however, almost entirely without exception, artificial mechanical means, and often unskilful violence, were used in extracting the fœtus." In reply to the query presented to him as to whether hemorrhage might be more commonly met with in very young than more aged animals, his language in substance is as has been already transcribed, and where in conclusion he further states, "However exhausted by parturient efforts, I never knew hemorrhage occur which could be supposed due to that state, and the attendant want of uterine contraction. A few ounces of blood are often lost at healthy parturition, and this comes entirely from the ruptured navel string, which you are aware we do not tie."

As proof of what he has here advanced, Mr. B. alludes to the fact that, amongst cows, the womb frequently becomes everted soon after the birth of their calves, and prior to the expulsion of the membranes, &c. "Before the uterus can be returned," adds he, "the membranes require to be detached from the numerous cotyledonous surfaces. In effecting this, a gray or rather milky-looking fluid appears on the uterine and fœtal points of separation; but unless unskilful violence be used, and presuming all to be healthy, I never saw any blood." "In the mare," says he, "I never saw a case of post-partum hemorrhage, unless, as I stated regarding the cow, it depended on actual rending or lesion of surface, not due to separation of membranes." The same intelligent and scientific practitioner favors us with the results of his experience in a case of cæsarean section, in which he operated upon a bitch in the month of August last. "The animal," he states, "was small, but the puppies large, on account of her having connection with a large dog. She had been in labour for two days, and being much exhausted, the pains were entirely ceased. On laying open the uterus, a dead fœtus was found impacted in the os uteri and swollen vagina; four other fœtuses were extracted alive, enclosed in their membranes, which, of course, required to be detached from the surface of the uterus. It immediately collapsed, but did not contract. The mother was preserved alive by means of stimulants for about four hours; the puppies sucked. She remained in a state of collapse, but still there was no contraction nor hemorrhage. On dissection, the womb was flaccid and collapsed; but making allowance for what had been removed, there did not seem to be any contraction. I carefully examined the placental surface: there was not any hemorrhage." Several similar cases, he informs us, were attended with the like results. As still farther tending to develop the physiological history of the gravid uterus amongst inferior animals, compared with woman, it has been deemed proper, along with the lengthened extracts already presented to the profession from the pen of this gentleman, to add the following very important paragraph. In reply to the query, Had he ever met with or known of any case where the placenta, was extended over the uterine orifice, he thus states:—"I never saw or heard of a case wherein the placenta was attached over the entrance of the womb in quadrupeds. In all the cases of parturition I have witnessed, hemorrhage before delivery has never been observed; and in the many cases of abortion in cows and mares which come under our notice, no discharge of blood ever occurs before the fœtus is expelled."—*Edinburgh Medical and Surgical Journal.*

